# KS-WNK1 augments the effects of dietary potassium intake on renal sodium chloride reabsorption

**DOI:** 10.1172/JCI195512

**Published:** 2025-08-01

**Authors:** Gerardo Gamba, David H. Ellison

**Affiliations:** 1 Molecular Physiology Unit, Instituto de Investigaciones Biomédicas, Universidad Nacional Autónoma de México and Instituto Nacional de Ciencias Médicas y Nutrición Salvador Zubirán, Tlalpan, Mexico City, Mexico.; 2 Division of Nephrology and Hypertension, Departments of Medicine and Chemical Physiology and Biochemistry, Oregon Health & Science University, Portland, Oregon, USA.

## Abstract

Clinically, potassium supplementation has been shown to lower blood pressure and reduce the risk of stroke through modulation of potassium excretion and sodium reabsorption. Hypokalemia activates the renal sodium chloride cotransporter (NCC) along the distal convoluted tubule (DCT), at least in part, through with-no-lysine 4 (WNK4) kinase and STE20/SPS1-related proline-alanine-rich protein kinase (SPAK) signaling. The DCT also expresses a kinase-deficient, kidney-specific form of WNK1 (KS-WNK1), but its role in NCC activation is unclear. In this issue of the *JCI*, Boyd-Shiwarski and colleagues found that KS-WNK1 enhanced the effects of potassium on NCC activation in vivo. Specifically, they showed that mice lacking KS-WNK1 did not respond as robustly to dietary challenge. Additionally, in vivo expression of a mutated KS-WNK1 disrupted WNK body, or biomolecular condensate, formation and renal function. These findings, along with those of previous studies, indicate that KS-WNK1 may regulate potassium homeostasis by increasing the kidney’s sensitivity to salt-dependent stress.

## Potassium intake and blood pressure

In nature, mammals are exposed to wide variations in potassium (K^+^) consumption, from little or no intake for several days to very high absorption following meat ingestion. Thus, the kidney requires powerful mechanisms to respond to these changes. The discovery of with-no-lysine (WNK) kinases in 2000 (1) and the subsequent demonstration that WNK kinase mutations can disrupt systemic potassium balance and blood pressure in humans (2) have improved our understanding of the pathways that modulate sodium and potassium excretion. During the ensuing 25 years, a new paradigm has developed, suggesting that low-potassium intake stimulates WNK4 to phosphorylate and activate the STE20/SPS1-related proline-alanine-rich protein kinase (SPAK, also known as serine/threonine kinase 39 [STK39]), which in turn phosphorylates and activates the renal sodium chloride cotransporter (NCC). NCC activation helps prevent potassium loss by increasing sodium reabsorption with chloride and limiting distal sodium delivery (3, 4).

A clear relationship exists between systemic blood pressure and potassium metabolism. In general, low potassium intake is associated with higher blood pressure and cardiovascular mortality (5). Large randomized clinical trials have also shown recently that potassium supplementation can reduce blood pressure and mortality (6). The molecular mechanisms behind this inverse relationship reside, at least in part, in the distal convoluted tubule (DCT) of the kidney (7). The activity of the NCC controls the amount of sodium delivered to the connecting tubule and collecting duct, which is required for the electrogenic reabsorption that allows potassium secretion. Thus, low extracellular potassium concentration activates the NCC to prevent urinary potassium loss; consequently, this process favors increased salt reabsorption and higher blood pressure. Data from patients with familial hyperkalemic hypertension (also called Gordon syndrome) have shown that the hyperkalemia and high blood pressure that these patients experience are associated with mutations that increase WNK signaling and can be successfully treated with thiazide diuretics (8).

The WNK-SPAK-NCC model of renal potassium regulation is now well accepted, but there are some aspects of this pathway that have remained controversial (9). WNK4 is the predominant WNK kinase in the DCT. However, WNK1, which is found ubiquitously throughout the body in its full-length form, has a shorter, kidney-specific isoform (KS-WNK1) that is almost exclusively expressed in the DCT (10, 11). This isoform is expressed through the use of an alternative promoter. It therefore lacks the first three exons of the full-length protein that encode the kinase domain and contains an alternative exon 4a with 30 unique amino acid residues. This unique sequence within KS-WNK1 appears to be crucial for the formation of WNK bodies — biomolecular condensates that promote NCC activation during hypokalemia (12). The role of KS-WNK1 in DCT physiology has thus far been unclear, with studies suggesting that it can activate or inhibit NCC (9). These disparate results led one group to design a study that would help resolve this confusion.

## KS-WNK1, plasma K^+^, and NCC activation

In this issue of the *JCI*, Boyd-Shiwarski et al. (13) present compelling evidence that KS-WNK1 can both activate NCC during low-potassium intake and limit its activity under high-potassium conditions. They used control mice (WT) and KS-WNK1–KO mice from both sexes to assess NCC activity in mice fed low, control, and high-potassium diets. Healthy mice can typically handle a high-potassium diet, so the diuretic amiloride was also given to a group of mice fed the high-potassium diet to generate hyperkalemia.

**Figure 1:**
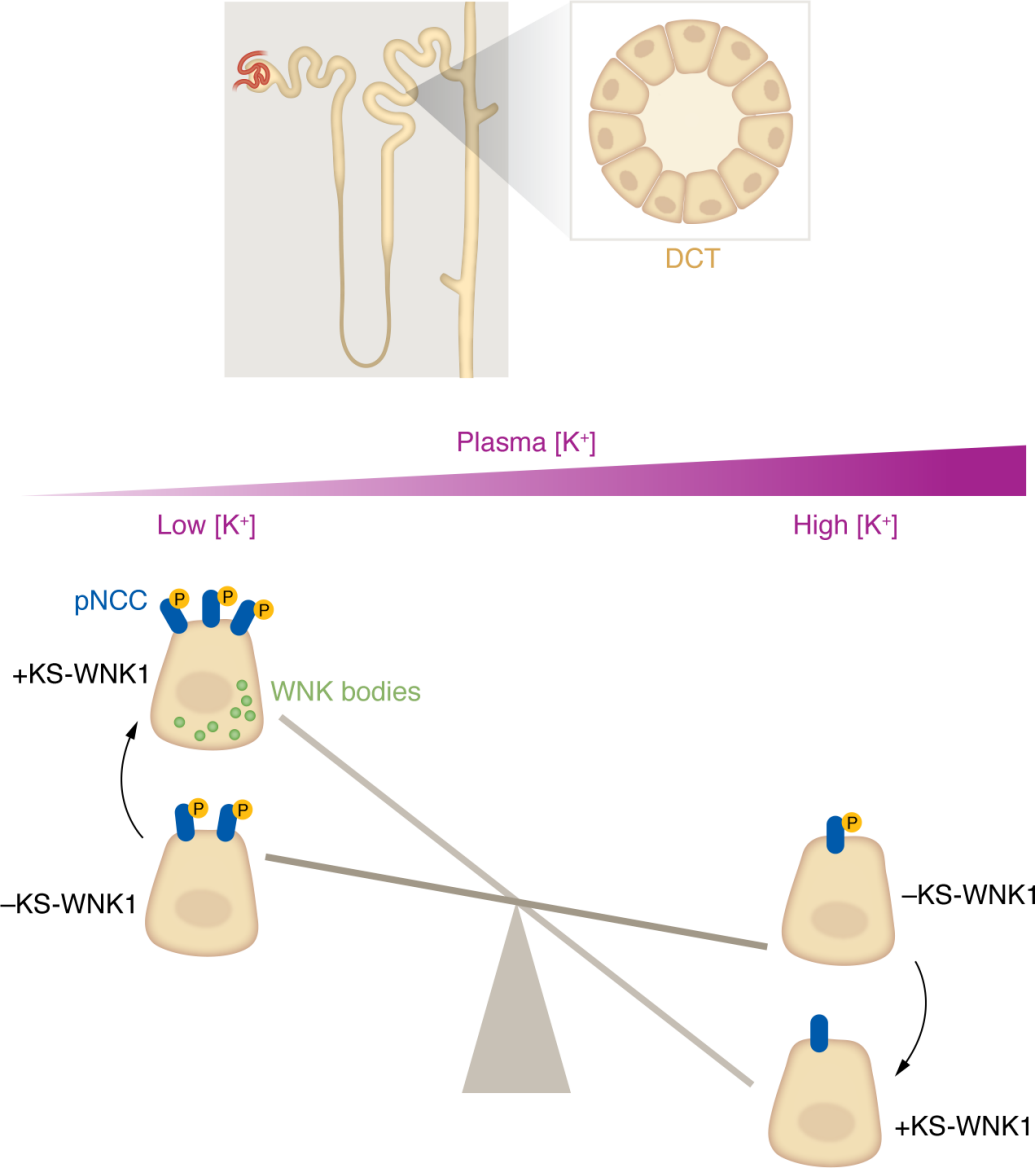
KS-WNK1 increases sensitivity of NCC activation to changes in plasma potassium concentration [K^+^]. When plasma [K^+^] is low, NCC is phosphorylated (pNCC) and activated through WNK/SPAK signaling to mitigate urinary potassium loss. This process is enhanced by KS-WNK1, an isoform necessary for the formation of WNK bodies, which are biomolecular condensates. When plasma [K^+^] is high, NCC activity and phosphorylation are reduced, and WNK bodies dissipate. When KS-WNK1 is absent, the effects of plasma [K^+^] on NCC activation are still present but less pronounced. Under these conditions, WNK bodies do not form at any plasma [K^+^].

No difference in NCC expression or phosphorylation (pNCC) was observed between WT and KS-WNK1–KO mice consuming a control diet. Strikingly, phosphorylation of NCC in KS-WNK1–KO kidneys was lower during hypokalemia and higher during hyperkalemia relative to the changes observed in WT mice. Thus, KS-WNK1–KO mice still appeared able to modulate pNCC in response to changes in blood potassium, but the response was dampened compared with that of WT mice (Figure 1). This result is consistent with other recent work that suggests that KS-WNK1 can amplify the response of NCC to changes in potassium intake (14). A possible mechanism for this effect derives from the observation that KS-WNK1 forms heterodimers with WNK4, which could promote WNK4 activation during low K^+^ intake and increase phosphorylation of NCC (15).

The authors next used clever mathematical modeling to reveal distinct differences in pNCC activity compared with blood potassium concentration based on the presence or absence of KS-WNK1 (13). Whereas the inverse response of pNCC to blood potassium was linear in KS-WNK1–KO mice, this was not true in WT mice. Instead, there was a breakpoint around a blood K^+^ of 5.6 mmol/L, which is considered the upper limit of the normal serum K^+^ concentration range. Phosphorylation and dephosphorylation of NCC below and above this limit, respectively, were much more efficient in WT than in KS-WNK1–KO mice, as evidenced by the sharper slopes of these curves.

The effect of KS-WNK1 on NCC activations was also linked to the formation of WNK bodies when blood potassium concentration was less than 4.0 mmol/L (Figure 1). WNK bodies or molecular condensates have been associated with activation of the WNK/SPAK pathway in cultured cells exposed to hypertonicity and in DCT cells in vivo in the presence of hypokalemia (3, 16–18). Consistent with this, it was previously shown that WNK bodies cannot form in DCT in the absence of KS-WNK1 (12).

Interestingly, although the effect of low-potassium diet on pNCC and SPAK phosphorylation (pSPAK) followed similar trends, the dephosphorylation of pNCC during high-potassium conditions was not associated with comparable changes in pSPAK. These results indicate that the effect of hyperkalemia on NCC dephosphorylation may be associated with an independent, unknown role for KS-WNK1, as has been previously suggested (14, 19).

## Role of WNK bodies in NCC activation

Boyd-Shiwarski and colleagues previously showed that KS-WNK1 is required for WNK body formation in mice exposed to low-potassium intake (12). However, to test whether WNK body formation itself is essential for full NCC activation, the group expressed a modified KS-WNK1 in mice. They had found that a unique stretch of cysteines within the KS-WNK1 amino-terminal domain was required for KS-WNK1 to form molecular condensates in cells (12). In this study, they introduced five glutamine mutations (5Q) into this region of KS-WNK1 in vivo to determine whether it could disrupt both WNK body formation and NCC activation (13). The results were positive but perhaps with some caveats.

The KS-WNK1 5Q mice that consumed a low K^+^ diet did form molecular condensates, but these were distinct from classical WNK bodies. The mutant mice had large, irregular molecular condensates that appeared to sequester pSPAK within them. The KS-WNK1 5Q mice also demonstrated more pronounced hypokalemia and less NCC activation. The authors argue that this suggests that well-formed WNK bodies are required to activate and send pSPAK to the apical membrane for phosphorylation and activation of NCC.

While this explanation appears consistent with the data presented, some additional aspects of the KS-WNK1 5Q phenotype suggest caution. This group previously reported that the KS-WNK1 5Q mutant did not form aggregates when expressed in cells but was instead diffusely distributed, as might be expected if the amino-terminal cysteines are necessary for condensate formation (12). However, molecular condensates were still observed in KS-WNK1 5Q mice (13). Another study showed that the KS-WNK1 5Q mutant failed to activate the NCC but was also insensitive to degradation by the ubiquitin ligase complex formed by CLU3/KHLH3 (20). Thus, the KS-WNK1 5Q mutant may accumulate abnormally in the DCT and impact the size of WNK bodies.

The effects KS-WNK1 5Q on NCC activity also appear to be sexually dimorphic. Male KS-WNK1 5Q mice exhibit more total NCC under control conditions, whereas female mice do not. In addition, while female KS-WNK1 5Q mice developed severe hypokalemia on a low K^+^ diet, male mice did not. Additionally, male KS-WNK1 5Q mice exhibited higher serum chloride concentration and lower bicarbonate, when consuming a control diet. Thus, while the KS-WNK1 5Q mutant failed to form condensates in cultured cells, the results are more mixed in vivo, suggesting that this mutation may have other effects. There is some evidence that females may need more robust mechanisms to preserve potassium homeostasis during physiological stresses such as pregnancy (21), but the mechanisms underlying the sex differences observed in KS-WNK1 mutant mice and their response to potassium changes will require careful future study to decipher them.

## Conclusions and future studies

Despite these caveats, the current results bring us a fuller understanding of how KS-WNK1 regulates the NCC response to dietary potassium intake. As a pseudokinase, KS-WNK1 promotes the formation of WNK bodies and association with other WNK kinase family members under hypokalemic conditions to augment SPAK and NCC activity. In contrast, KS-WNK1 also appears to enhance dephosphorylation of NCC in hyperkalemic settings, but this seems to occur independently of SPAK. KS-WNK1 therefore allows mice, and people, to adapt rapidly to a wider range of physiological changes and the challenges of episodic potassium consumption. Given the known effect of WNK signaling on hypertension, these findings may also impact clinical management of blood potassium and blood pressure through reevaluation of blood chemistry references ranges, diet recommendations, and use of hypertensive therapeutics.

## Version 1 08/01/2025

Electronic publication
